# Histopathological evaluation of pulp response to portland cement compared to MTA after primary canines pulpotomy (in vivo study)

**DOI:** 10.1038/s41405-022-00121-9

**Published:** 2022-10-01

**Authors:** Hasan Alzoubi, Nada Bshara, Ahmad AL-Manadili

**Affiliations:** 1 Department of Pediatric Dentistry, College of Dentistry, Damascus University, Damascus, Syria; 2 Department Oral Histopathology, College of Dentistry, Damascus University, Damascus, Syria

**Keywords:** Dental biomaterials, Mineral trioxide aggregate

## Abstract

**Introduction:**

ECC (Early childhood caries) is very common in children. Because of the small size of primary anterior teeth, endodontic exposures occur early. Pulp tissue response after pulpotomy of primary anterior teeth by both MTA and Portland Cement is very important when pulp exposures occur in these teeth.

**Aim:**

This study aimed to evaluate in vivo pulp tissue responses after the primary canines pulpotomy with either White Portland Cement (WPC) or White Mineral Trioxide Aggregate (WMTA), by histopathological analysis.

**Materials and methods:**

The study included 30 primary canines in 21 healthy children aged 6–9 years old and it was classified into 2 groups according to the material. Group 1: included 15 teeth capped by White Portland Cement, and Group 2: included 15 teeth capped by white MTA. The dentine bridge formation, soft Tissue Organization, tissue fibrosis, formed dentin bridge thickness, pulp calcifications, hemorrhage in the pulp tissue, and deposition of new dentin on the inner surface of the dentin at 3 months periods were recorded.

**Results:**

Data were analyzed statistically; the Mann–Whitney U test was performed for the assessment of histopathological criteria. Descriptive statistics were performed for the analysis of participant properties. Histopathological analysis showed complete dentin bridge formation and normal soft tissue organization for both materials. Statistical analysis showed no significant differences in dentine bridge formation (*P* value = 0.213), soft Tissue Organization (*P* value = 0.339), tissue fibrosis (*P* value = 0.079), formed dentin bridge thickness (*P* value = 0.139), pulp calcifications (*P* value = 0.581), hemorrhage in the pulp tissue (*P* value = 0.117), and deposition of new dentin (*P* value = 0.097), during the observation period.

**Conclusions:**

Within the limitation of the current study WPC was similar to WMTA in terms of histological criteria so PC may serve as a good alternative to MTA in primary teeth pulpotomy.

## Introduction

Primary pulp tissue is a specialized connective tissue of mesenchymal origin that forms during the sixth week of uterine life. The pulp tissue is composed of loose fibrous connective tissue, Schwann cells, red blood cells, and stem cells. The shape of the pulp tissue matches the general anatomical shape of the tooth [1].

Primary teeth are important for the normal growth and development of a child. The early loss of anterior teeth negatively affects the child’s occlusion, psychosocial development, and facial esthetics. Primary teeth show morphological differences, which in turn reflect different functional roles and help in the process of chewing food [2].

Early loss of anterior teeth can affect the initiation or prolongation of common childhood oral habits such as pacifier use, finger sucking, and tongue thrusting [3]. Studies have shown that children with a normal dental appearance appear better esthetically, are more acceptable as friends, are more intelligent, and exhibit better social behavior [4].

Vital pulp therapy has spread widely in recent years due to its high success rates and its preference by dentists in cases of traumatic pulp exposure or during caries removal, in addition to its low cost compared to root canal treatment [5]. Pulpotomy is defined as a procedure based on whether the root pulp tissue is healthy or curable after the surgical removal of the affected coronal pulp [6].

Portland cement is a fine powder that consists of the following: calcium oxide (including free calcium oxide and gypsum), silicon oxide, aluminum oxide, iron oxide, magnesium oxide, titanium oxide, sulfur trioxide, and alkali metal oxides [7].

Despite the high success rates of pulpotomy by using many materials for primary molars, which ranged from 83% to 100%, there is a lack of literature that shows the results of pulpotomy in primary anterior teeth (incisors and canines) [8, 9].

The poor prognosis of pulpotomy in primary anterior teeth can be attributed to the historically poor sealability of pulpotomy materials. With the advent of biocompatibility materials such as MTA/ Biodentine/Portland Cement, the success rates of pulpotomy have reached 100%, so a change in the direction of the materials used in pulpotomy can lead to a change in the choice of treatment by the dentist [10, 11].

In Alzoubi et al study, primary anterior teeth pulpotomy achieved a 100% clinical and radiographical success rate during a 12-month observation period [12].

Because of the similarities between the properties of MTA and Portland cement and its encouraging clinical and radiographic success rate in primary anterior teeth pulpotomy, this study hypothesizes the possibility of finding differences in pulp tissue response after applying both MTA and PC as pulpotomy agents in primary canines.

Therefore, the aim of this study was to evaluate the response of pulpotomized primary dental pulp canines in pediatric patient to White Portland cement and White MTA, the evaluation was carried out by histopathological analysis.

## Materials and methods

### Ethical considerations

The study protocol was approved by the Scientific research and Postgraduate Board of Damascus University, Ethics Committee, Damascus University, Syria (IRB No. UDDS-1786-04032019/SRC-1450). The study protocol was also can be accessed at clinicaltrials.gov (NCT04634123). A detailed information sheet in simple nontechnical language was provided in advance, and parents/guardians were requested to sign an informed consent. The patients and parents were blinded by not being provided any information about the treatment group to which they were selected.

### Sample size and power calculation

The sample size was determined using a sample size calculation program (PS Power and Sample Size Calculation Program, Version 3.0.43). Sample size calculation produced a required sample size of 13 primary canines per group to detect a significant difference (significance level of 5% and a power of 90%, effect size = 1.19). To compensate for drop-out rate of 20%, the number was increased to at least 2 primary canines per group were added to each group with a total sample size of 30 primary canines.

### Study population and inclusion criteria

A total of 30 primary canines in 21 patients were assessed for the study and invited to participate in the investigation following the inclusion criteria: primary canines indicated for extraction for orthodontic reasons (serial extraction and interceptive treatment), vital pulp with no fistula or abscess, absence of internal or external root resorption at the radiographic examination, and physiological root resorption no more than the apical third. Exclusion criteria were related to the presence of systemic pathology and history of allergic reaction to local anesthetics or some of the constituents of the dressing materials.

### Randomization

**Fig. 1:**
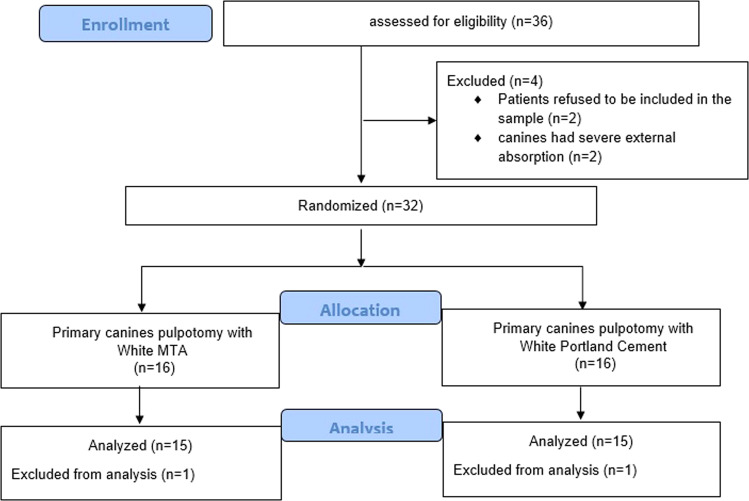
CONSORT flow diagram.

The histopathological sample was studied according to CONSORT criteria and was randomly distributed at http://www.randomization.com into two groups (Fig. 1): Group (A) (represented the experimental group which was treated with White Portland Cement) and Group (B) (represented the control group which was treated with the White MTA).

A double-blinded was also adopted in this study so that both the patient and the examiner would not know about the applied material (The examiner was blinded by giving him numbered slides without prior knowledge of which group the slide belonged to).

Histological evaluation was carried out with the help of two resident histopathologists. In case of disagreement between residents, a third assessor was used and the majority opinion was adopted.

### Treatment procedure

All dental treatments were provided at Damascus University-Faculty of Dentistry-Department of pediatric dentistry with local anesthesia and rubber dam isolation. The coronal pulp was amputated to a depth of approximately 2 millimeters below the free gingival margin at a high speed. hemostasis was achieved by applying pressure with a sterilized cotton ball moistened with saline. If hemostasis was not achieved, the tooth would be eliminated from the study. After hemostasis, WMTA (ProRoot® MTA Root Repair Material, Dentsply, Maillefer) or WPC (Aalborg, Sinai, Egypt) were applied to the amputated pulp surface to a thickness of not less than 1 millimeter using an amalgam carrier. The pulp chamber was then sealed with glass ionomer cement (Fuji IX®, GC Corporation, Tokyo, Japan). The tooth was restored with acid etch resin composite (Filtek Z250®, 3 M ESPE, St. Paul, MN, USA) immediately. Teeth were extracted after 3 months and were prepared for histological evaluation.

### Outcome assessment

Extracted teeth were fixed immediately in 10% neutral formalin solution for 72 h. Afterward, the teeth were placed in sodium citrate buffered formic acid for demineralization. Subsequently, each tooth was embedded in paraffin wax and 5 µm-thick serial sections were obtained and stained with hematoxylin and eosin. The histological evaluation was made under a light microscope (Carl Zeiss, Oberkachen, Germany), the primary outcomes (Dentine bridge formation, deposition of new dentin on the inner surface of the dentin, soft Tissue Organization, and Pulp calcifications) and secondary outcomes (Formed dentin bridge thickness, Fibrosis, and hemorrhage in the pulp tissue) were evaluated based on the following scores:

a) Dentine bridge formation score:

score 0 = There is no evidence of its formation in any section, score 1 = The dentin bridge begins to form, score 2 = dentin bridge formation but is not completely completed, score 3 = complete dentin bridge formation.

b) Deposition of new dentin on the inner surface of the dentin:

score 0 = There is no extra dentin deposition anywhere in the pulp dentin complex, score 1 = A thin strip of neo dentin over the entire inner surface of the dentin, score 2 = A thick strip of fresh dentin all over the inner surface of the dentin.

c) Formed dentin bridge thickness:

score 0 =>  0.25 mm, score 1 = 0.1–0.25 mm, score 2 < = 0.1 mm, score 3 = Partial or complete absence of the dentinal bridge.

The thickness of the dentinal bridge will be measured at the thickest, medium, and thinnest points, and the mean of these points will be based on the value of the thickness of the formed dentinal bridge

d) Pulp calcifications:

score 0 = No calcifications, score 1 = single small calcification (diameter smaller than 200 μm), score 2 = multiple small calcifications, score 3 = single large calcification, score 4 = Multiple large calcifications

e) Soft Tissue Organization:

score 0 = Normal morphological tissue structure in the pulp at the site of pulpotomy or under the dentin bridge and all the pulpal tissue with no necrosis., score 1 = A slight deficiency in the normal morphological structure of the pulp located in the superficial layers of the pulp or under dentin bridge with a normal central residual pulp, score 2 = A moderate or wide deficiency in the morphological structure of pulp tissue is deeper than the superficial layers of the pulp., score 3 = Pulp necrosis.

f) Fibrosis score:

Score 0 = no fibrosis, score 1 = Mild fibrosis (thin layer of collagen fibers), score 2 = Moderate fibrosis, score 3 = Sever fibrosis (thick layer of collagen fibers).

g) hemorrhage in the pulp tissue:

Score 0 = No hemorrhage, score 1 = Slight hemorrhage (next to dentin bridge or area of pulp exposure only), score 2 = Moderate hemorrhage (in one-third or or in the midpulp)., score 3 = Heavy hemorrhage (all of the pulp).

### Statistical analyses

All data are expressed as the mean + standard deviation (SD). Comparisons between each experimental group were performed using the Mann–Whitney U test. The statistical significance was defined as *p* < 0.05.

## Results

**Fig. 2:**
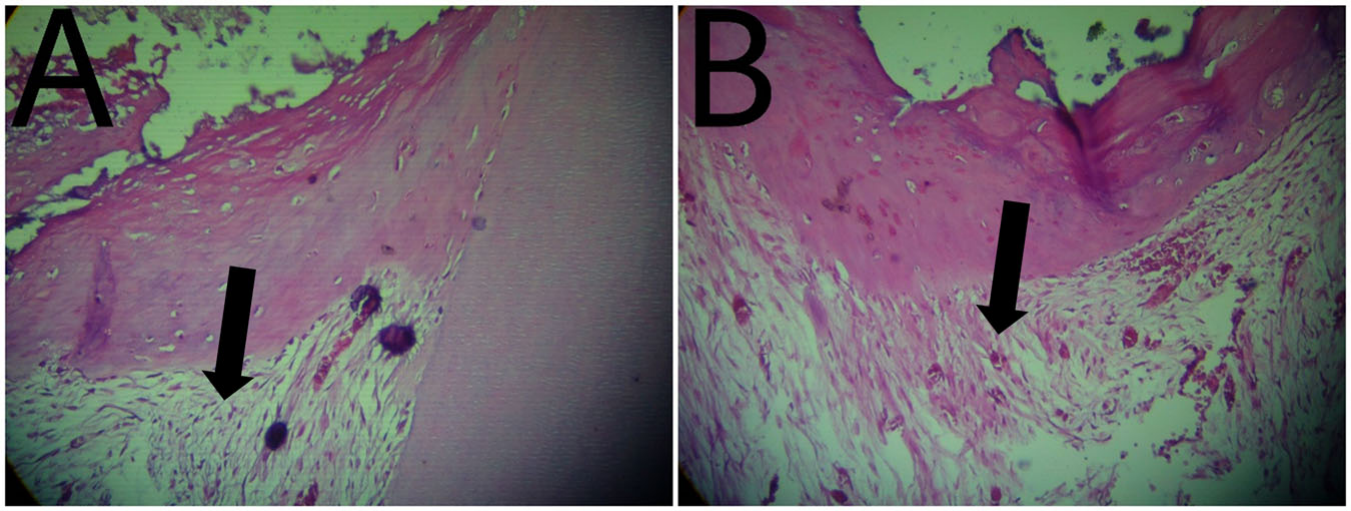
The arrow points to soft tissue organization. **A** Photomicrograph of dental pulp capped with Portland Cement. **B** Photomicrograph of dental pulp capped with MTA.

The study sample consisted of 30 cases of pulpotomy performed for 30 primary canines in 21 children of both sexes, their ages ranged between 6 and 9 years, with an average of 7.9 ± 0.9, and the Mann–Whitney U test was performed to compare the two groups. The results of the Histopathological criteria were:

Soft tissue organization: at the third month of follow up soft tissue organization of each material was the same, in both groups, 80% of specimens showed normal tissue morphology and 20% of specimens showed a lack of normal tissue morphology (Table 1 and Figs. 2, 6, 7).

**Table. 1:** Results of histopathological criteria.

Histopathological criteria	Score	White portland cement	White MTA	U-value	*P* value
Soft tissue organization	_Score 0_	_12 (80%)_	_12 (80%)_	_12.5_	_0.339_
	_Score 1_	_3 (20%)_	_3 (20%)_		
	_Score 2_	_0 (0%)_	_0 (0%)_		
	_Score 3_	_0 (0%)_	_0 (0%)_		
Fibrosis	_Score 0_	_3 (20%)_	_6 (40%)_	_8.5_	_0.079_
	_Score 1_	_9 (60%)_	_9 (60%)_		
	_Score 2_	_3 (20%)_	_0 (0%)_		
	_Score 3_	_0 (0%)_	_0 (0%)_		
Dentin bridge formation	_Score 0_	_0 (0%)_	_0 (0%)_	_10.0_	_0.213_
	_Score 1_	_0 (0%)_	_0 (0%)_		
	_Score 2_	_6 (40%)_	_3 (20%)_		
	_Score 3_	_9 (60%)_	_12 (80%)_		
Dentin bridge thickness	_Score 0_	_0 (0%)_	_3 (20%)_	_12.5_	_0.139_
	_Score 1_	_15 (100%)_	_9 (60%)_		
	_Score 2_	_0 (0%)_	_3 (20%)_		
	_Score 3_	_0 (0%)_	_0 (0%)_		
Pulp calcifications	_Score 0_	_12 (80%)_	_12 (80%)_	_12.0_	_0.581_
	_Score 1_	_0 (0%)_	_0 (0%)_		
	_Score 2_	_0 (0%)_	_3 (20%)_		
	_Score 3_	_3 (20%)_	_0 (0%)_		
Hemorrhage in the pulp tissue	_Score 0_	_15 (100%)_	_12 (80%)_	_10.0_	_0.117_
	_Score 1_	_0 (0%)_	_3 (20%)_		
	_Score 2_	_0 (0%)_	_0 (0%)_		
	_Score 3_	_0 (0%)_	_0 (0%)_		
Deposition of new dentin on the inner surface of the dentin	_Score 0_	_0 (0%)_	_0 (0%)_	_10.0_	_0.097_
	_Score 1_	_15 (100%)_	_12 (80%)_		
	_Score 2_	_0 (0%)_	_3 (20%)_

**Fig. 3:**
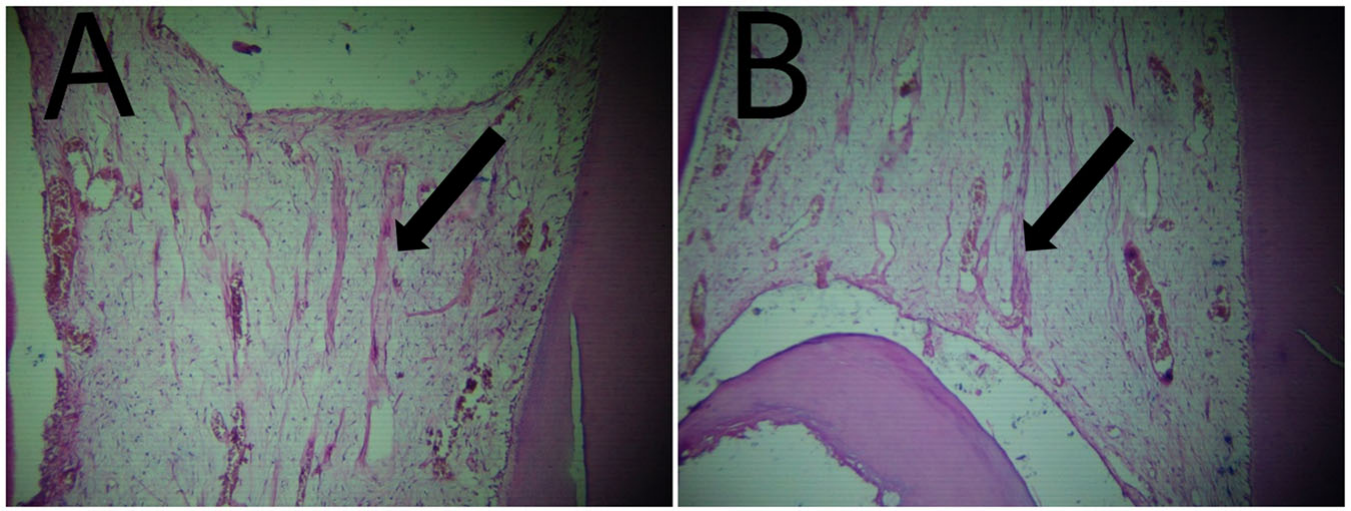
The arrow points to tissue fibrosis. **A** Photomicrograph of dental pulp capped with Portland Cement. **B** Photomicrograph of dental pulp capped with MTA.

Fibrosis: at the third month of follow up 20% of specimens in White Portland Cement specimens showed no fibrosis, 60% of specimens showed Mild fibrosis and 20% showed Moderate fibrosis. While in White MTA 40% of specimens showed no fibrosis and 60% of specimens showed Mild fibrosis (Table 1 and Figs. 3, 6, 7).

**Fig. 4:**
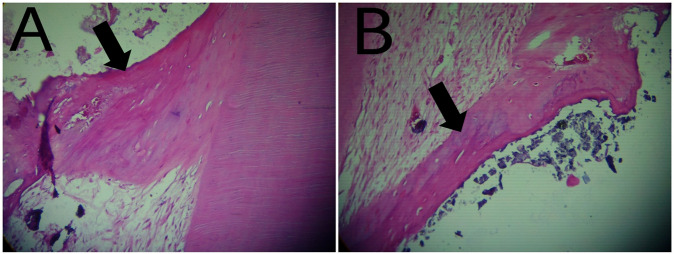
The arrow points to dentin bridge formation. **A** Photomicrograph of dental pulp capped with Portland Cement. **B** Photomicrograph of dental pulp capped with MTA.

Hard tissue deposition: At the third month of follow up 40% of specimens in White Portland Cement specimens showed moderate hard tissue deposition beneath the exposed area and 60% of specimens showed Heavy hard tissue deposition beneath the exposed area. While in White MTA 20% of specimens showed moderate hard tissue deposition beneath the exposed area and 80% of specimens showed Heavy hard tissue deposition beneath the exposed area (Table 1 and Figs. 4, 6, 7).

Dentin bridge thickness: at the third month of follow up 100% of specimens in White Portland Cement specimens showed 0.1–0.25 mm dentin bridge thickness. While in White MTA 20% specimens => 0.25 mm dentin bridge thickness, 60% specimens showed 0.1–0.25 mm dentin bridge thickness and 20% showed < = 0.1 mm dentin bridge thickness (Table 1 and Figs. 6, 7).

**Fig. 5:**
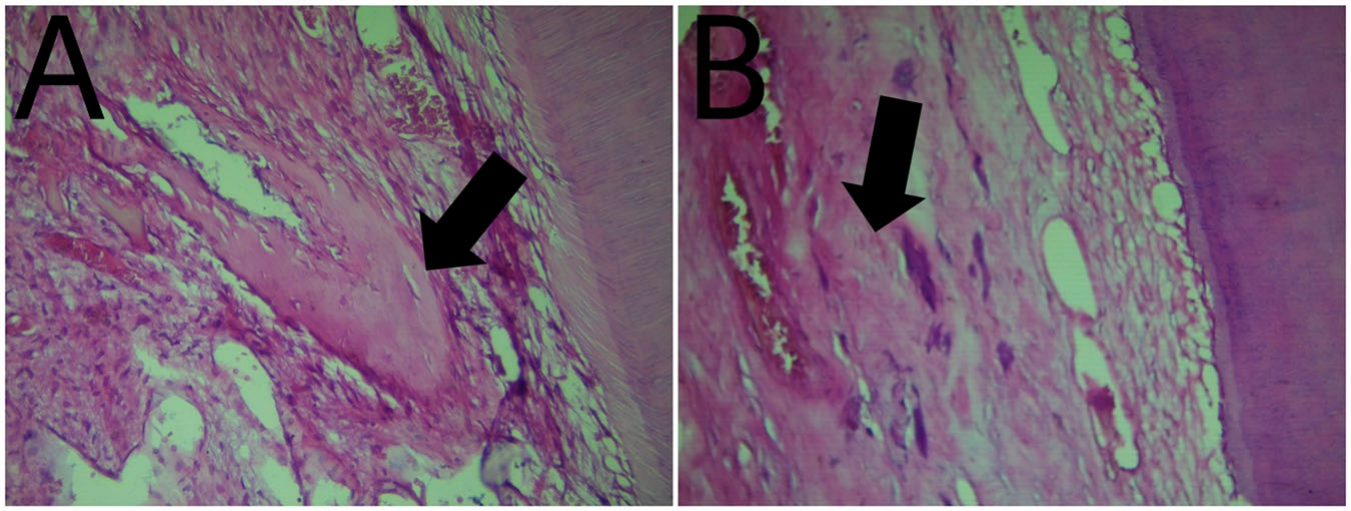
The arrow points to pulp calcifications. **A** Photomicrograph of dental pulp capped with Portland Cement. **B** Photomicrograph of dental pulp capped with MTA.

**Fig. 6:**
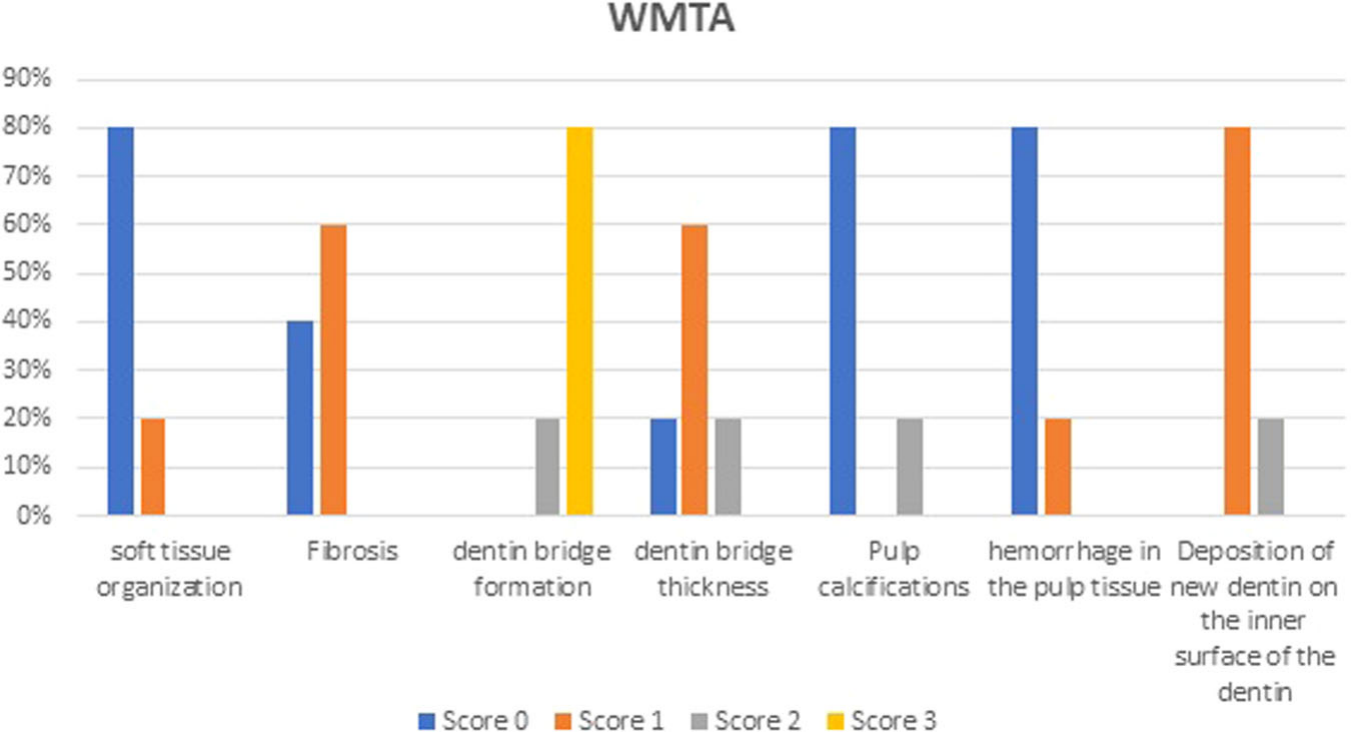
The average percentage of histopathological pulp response to WMTA.

**Fig. 7:**
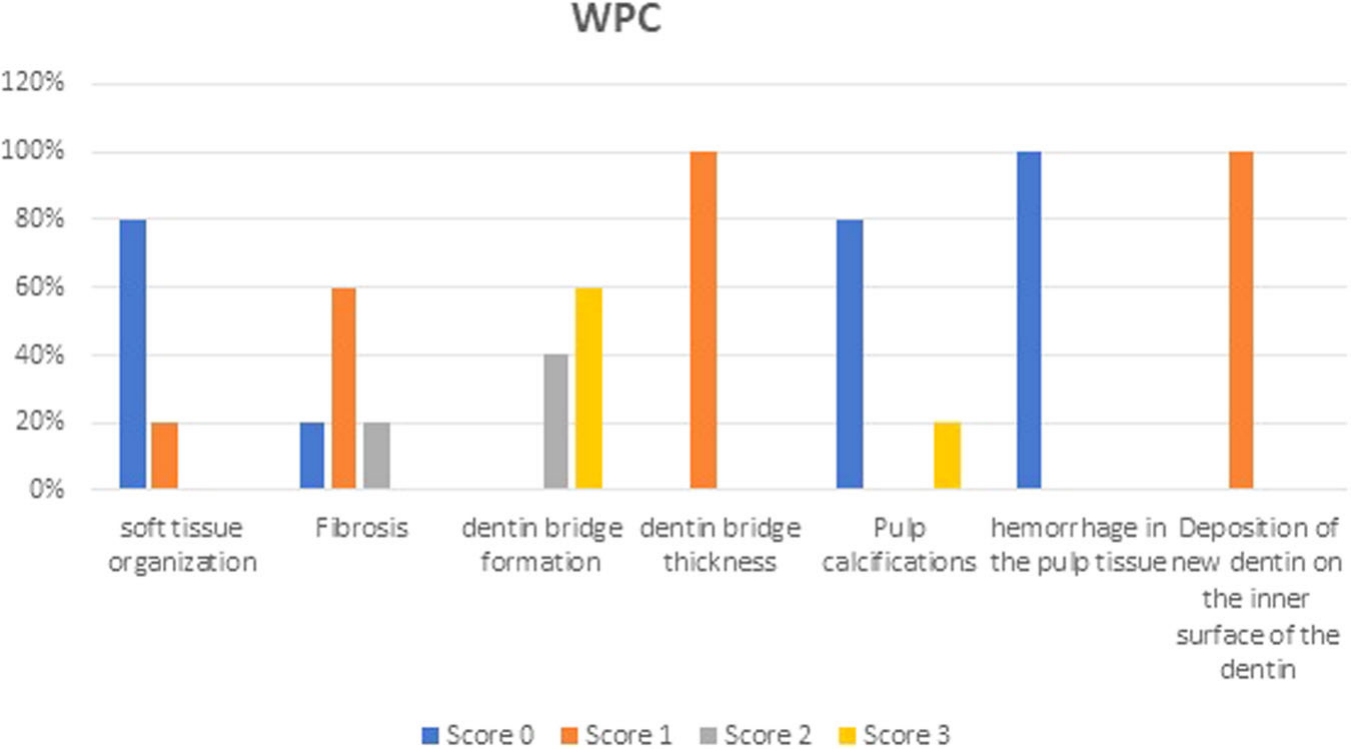
The average percentage of histopathological pulp response to WPC.

Calcifications: at the third month of follow up 80% of specimens in White Portland Cement specimens showed no calcifications and 20% of specimens showed Multiple large calcifications. While in White MTA 80% of specimens showed no calcifications and 20% single large calcification (Table 1 and Figs. 5–7).

Hemorrhage in the pulp tissue: at the third month of follow up 100% of specimens in White Portland Cement specimens showed no hemorrhage. While in White MTA 80% of specimens showed no hemorrhage and 20% slight hemorrhage (Table 1 and Figs. 6, 7).

Deposition of new dentin on the inner surface of the dentin: at the third month of follow up 100% of specimens in White Portland Cement specimens showed a thin strip of neo dentin over the entire inner surface of the dentin. While in White MTA 80% of specimens have a thin strip of neo dentin over the entire inner surface of the dentin and 20% = A thick strip of fresh dentin all over the inner surface of the dentin (Table 1 and Figs. 6, 7).

## Discussion

Pulpotomy can be performed with different materials based on their biocompatibility, sealing capacity, and antimicrobial efficacy. Portland cement and (MTA) are the most recently proposed biologically active materials [13], so the current study was conducted to compare the response of the dental pulp in pulpotomy primary canines by Portland cement compared with MTA.

The results of studies comparing MTA with Portland cement in terms of their composition showed that these two materials are almost identical[14]. MTA and PC consist of tricalcium silicate, tricalcium aluminate, calcium silicate, and tetracalcium aluminoferrite that are mixed with water to form calcium hydroxide [15].

Histopathological evaluation was performed to report the soft Tissue Organization, tissue fibrosis formed dentin bridge thickness and pulp calcifications, as well as dentine bridge formation using Hematoxylin, Eosin stains [16]. Therefore, the extracted teeth were evaluated after 3 months to observe the histopathological changes in tested materials.

PC has been found to promote a layer of “bone-like” hydroxyapatite which underpins its ability to integrate with dental tissue called dentin bridge. There was no statistically significant difference between groups in terms of dentine bridge formation and this finding goes by Holland et al. and Sayed et al. [17–19].

Hard tissue barrier was a greater finding in most of the cases in PC and MTA groups. The biological reaction of the pulp to PC was good and no significant difference was seen as the mechanism of action of MTA and PC are similar. Both materials have calcium oxide that forms calcium hydroxide when mixed with water, the reaction of the calcium from calcium hydroxide with the carbon dioxide from the pulp tissue produces calcite crystals [20]. This is the initial step of a hard tissue barrier formation, also known as dentin bridge [21].

All cases showed dentin bridge formation after three months of observation. Complete dentin bridge formation was observed in 60% of the WPC group and 80% of the WMTA group. The results of this study are in agreement with the study of Menezes et al. [22] and Bhagat et al. [23].

Moreover, there was no statistically significant difference between groups in terms of tissue fibrosis between the groups. An increase in the quality of fibrosis was observed after 3 months of application of Portland cement and MTA. A finding that goes by Tran et al. [24] and Sayed et al. [19].

Both Portland cement and MTA initially cause superficial death upon contact with the connective tissue of the pulp due to the high alkalinity of these materials, with a pH of approximately 9-10, and this was observed in all studied samples and this is and this is consistent with Camilleri et al. [25] and Barbosa et al. [26].

As for the soft tissue organism, likewise, there were no statistically significant differences between the two groups. Where the pulp tissue cells were normal and did not show any signs of pulp necrosis this finding goes by Barbosa et al. [26] and Sayed et al. [19]. While the results of this study did not agree with the study of Negm et al study and this could be attributed to its addition of bismuth oxide to the composition of Portland cement [27].

In addition, there was no statistically significant difference between the two groups (PC/MTA) in terms of fibrosis. An increase in fibrosis quality was observed after 3 months of application of PC and MTA and this result is in agreement with Tran et al. [24] and Sayed et al. [19].

PC Portland cement is less expensive and available compared to MTA, which may make it preferred by many dentists for clinical use, especially after achieving encouraging clinical, radiographical, and histological results for its application, and this is what many researchers have gone to [28–30].

## Conclusion

This study concluded the suitability of both WMTA and WPC as pulp capping materials in the pulpotomy of primary canines, because both materials behaved histologically similar regarding soft tissue organization, tissue fibrosis, dentin bridge formation, and pulp calcifications.

## References

[1] Nanci A. *Ten Cate’s Oral Histology-e-book: development, structure, and function*. 2017: Elsevier Health Sciences

[2] Clarke M, Locker D, Berall G, Pencharz P, Kenny DJ, Judd P (2006). Malnourishment in a population of young children with severe early childhood caries. Pediatr Dent.

[3] Holan G, Needleman HL (2014). Premature loss of primary anterior teeth due to trauma–potential short‐and long‐term sequelae. Dent Traumatol..

[4] Kapur A, Chawla H, Goyal A, Gaube K (2005). An esthetic point of view in very young children. J Clin Pediatr Dent..

[5] Parirokh M, Torabinejad M (2010). Mineral trioxide aggregate: a comprehensive literature review—part III: clinical applications, drawbacks, and mechanism of action. J Endod.

[6] Fuks AB, Kupietzky A, Guelmann M. Pulp therapy for the primary dentition, in *Pediatr Dent* 2019, Elsevier. 329-51.e1. 10.1016/B978-0-323-60826-8.00023-7.

[7] Islam I, Chng H, Yap A (2006). X‐ray diffraction analysis of mineral trioxide aggregate and Portland cement. Int Endod J.

[8] Fuks AB (2008). Vital pulp therapy with new materials for primary teeth: new directions and treatment perspectives. J Endod.

[9] Huth K, Paschos E, Hajek-Al-Khatar N, Hollweck R, Crispin A, Hickel R (2005). Effectiveness of 4 pulpotomy techniques—randomized controlled trial. J Dent Res.

[10] Agamy HA, Bakry NS, Mounir MM, Avery DR (2004). Comparison of mineral trioxide aggregate and formocresol as pulp-capping agents in pulpotomized primary teeth. Pediatr Dent.

[11] Mettlach SE, Zealand CM, Botero TM, Boynton JR, Majewski RF, Hu JC (2013). Comparison of mineral trioxide aggregate and diluted formocresol in pulpotomized human primary molars: 42-month follow-up and survival analysis. Pediatr Dent.

[12] Hasan Alzoubi NB, Imad K, Tamara K, Saleh A, Leen D (2021). Clinical and radiographic evaluation of using white portland cement and MTA in pulpotomy primary anterior teeth: a randomized, split-mouth, controlled clinical trial with 12 months follow-U. Int J Dent Oral Sci..

[13] Taha N, Ahmad M, Ghanim AJIEJ (2017). Assessment of mineral trioxide aggregate pulpotomy in mature permanent teeth with carious exposures. Int Endod J.

[14] Steffen R, Van Waes HJEAOPD (2009). Understanding mineral trioxide aggregate/Portlandcement: a review of literature and background factors. Eur Arch Paediatr Dent.

[15] Parirokh M, Torabinejad MJJOE (2010). Mineral trioxide aggregate: a comprehensive literature review—part I: chemical, physical, and antibacterial properties. J Endod.

[16] Bakhtiar H, Aminishakib P, Ellini MR, Mosavi F, Abedi F, Esmailian S (2018). Dental pulp response to RetroMTA after partial pulpotomy in permanent human teeth. J Endod.

[17] Holland R, Souza de V, Murata SS, Nery MJ, Bernabé PF, Otoboni Filho JA, et al. Healing process of dog dental pulp after pulpotomy and pulp covering with mineral trioxide aggregate or Portland cement. Braz Dent J. 2001;12:109–13. 11445912

[18] Holland R, de Souza V, Nery MJ, Faraco Júnior IM, Bernabé PF, Otoboni Filho JA (2001). Reaction of rat connective tissue to implanted dentin tube filled with mineral trioxide aggregate, Portland cement or calcium hydroxide. Braz. Dent. J..

[19] Sayed MM, Khattab N, Ahmed W (2018). Histopathological and histochemical evaluation of pulpal response to biodentine compared to portland cement in pulpotomized dogs’ teeth. EC Dent Sci.

[20] Seux D, Couble ML, Hartmann DJ, Gauthier JP, Magloire H (1991). Odontoblast-like cytodifferentiation of human dental pulp cells in vitro in the presence of a calcium hydroxide-containing cement. Arch Oral Biol.

[21] Takita T, Hayashi M, Takeichi O, Ogiso B, Suzuki N, Otsuka K (2006). Effect of mineral trioxide aggregate on proliferation of cultured human dental pulp cells. Int Endod J.

[22] Menezes R, Bramante CM, Garcia RB, Letra A, Carvalho VGG, Carneiro E (2004). Microscopic analysis of dog dental pulp after pulpotomy and pulp protection with mineral trioxide aggregate and white Portland cement. J Appl Oral Sci.

[23] Bhagat D, Sunder RK, Devendrappa SN, Vanka A, Choudaha N (2016). A comparative evaluation of ProRoot mineral trioxide aggregate and Portland cement as a pulpotomy medicament. J Indian Soc Pedod Prev Dent.

[24] Tran X, Gorin C, Willig C, Baroukh B, Pellat B, Decup F (2012). Effect of a calcium-silicate-based restorative cement on pulp repair. J Dent Res.

[25] Camilleri J, Montesin F, Silvio LD, Pitt Ford TR (2005). The chemical constitution and biocompatibility of accelerated Portland cement for endodontic use. Int Endod J.

[26] Barbosa AVH, dos Santos Junior VE, Martins MM, Ferreira LS, Sobral APV (2018). Human pulp pesponse to Portland cement and MTA. Rev Odonto Cienc.

[27] Negm AM, Hassanien EE, Abu-Seida AM, Nagy MM (2017). Biological evaluation of a new pulp capping material developed from Portland cement. Exp Toxicol Pathol.

[28] Chakraborty A (2015). Will Portland cement be a cheaper alternative to mineral trioxide aggregate in clinical use?: A comprehensive review of literature.. Int J Contemp Dent Med Rev.

[29] Reddy NV, Srujana P, Daneswari V, Konyala HR, Mareddy AR, Mohammad N (2019). Sealing ability of MTA vs Portland cement in the repair of furcal perforations of primary molars: a dye extraction leakage model—an in vitro study. Int J Clin Pediatr Dent..

[30] Meslmani W, Kouchaji C, Rekab S, Fakher MAA, Al Nerabieah Z (2020). The efficacy of Portland cement as a pulpotomy agent in deciduous teeth. Pediatr Dent.

